# A Canadian Perspective: Monoclonal Antibodies for Pre- and Post-Exposure Protection from COVID-19 in Vulnerable Patients with Hematological Malignancies

**DOI:** 10.3390/curroncol29060315

**Published:** 2022-05-31

**Authors:** Carolyn Owen, Sue Robinson, Anna Christofides, Laurie H. Sehn

**Affiliations:** 1 Division of Hematology and Hematological Malignancies, University of Calgary and Foothills Medical Centre, Calgary, AB T2N 4Z6, Canada; 2 Division of Hematology, Dalhousie University, Halifax, NS B3H 4R2, Canada; sue.robinson@nshealth.ca; 3 IMPACT Medicom Inc., Toronto, ON M6S 3K2, Canada; anna@impactmedicom.com; 4 BC Cancer Centre for Lymphoid Cancer, and The University of British Columbia, Vancouver, BC V6T 1Z4, Canada; lsehn@bccancer.bc.ca

**Keywords:** COVID-19, coronavirus, hematology, malignancy

## Abstract

Patients with hematological malignancies have an increased risk of serious outcomes following COVID-19 infection, suggesting broader protection is needed beyond vaccination. Monoclonal antibodies such as sotrovimab, casirivimab–imdevimab, and bamlanivimab have provided valuable options for the treatment of COVID-19 disease. More recently, monoclonal antibodies have been examined for the prevention of COVID-19 infection. The monoclonal antibody combination, tixagevimab–cilgavimab, was recently approved by Health Canada as pre-exposure prophylaxis against COVID-19 in individuals who are immunocompromised or where vaccination is not recommended. Prophylactic approaches such as the use of tixagevimab–cilgavimab, in addition to COVID-19 vaccination, may provide additional protection for patients with hematological malignancies who are at greater risk of serious outcomes from COVID-19 infection.

## 1. Introduction

Since the COVID-19 pandemic was first reported in China in December 2019, cases have exploded globally, with a total of 3.3 million cases and 36,630 deaths reported in Canada as of 2 March 2022 [1]. Although people of all ages are at risk of infection, the probability of more serious disease is greater in people with chronic medical conditions, who are 60 years or older, or who are living in nursing homes or long-term care facilities [2]. People who are immunocompromised are one such group with an increased risk of serious outcomes following COVID-19 infection [3]. Immunocompromising conditions are defined as those that suppress humoral or cellular immunity as a result of health conditions or medications [4]. Based on data from Statistics Canada, about 14% of Canadians fit into this higher-risk immunocompromised category [5]. Examples of patients with immunocompromising conditions include [6]:

- Active treatment for solid tumor and hematologic malignancies;
- Receipt of a solid-organ transplant and taking immunosuppressive therapy;
- Chimeric antigen receptor (CAR) T-cell therapy or hematopoietic stem cell transplant (HSCT);
- Moderate or severe primary immunodeficiency (e.g., DiGeorge syndrome, Wiskott–Aldrich syndrome);
- Advanced or untreated human immunodeficiency virus (HIV) infection.

In addition to having an increased risk of severe outcomes from COVID infection, studies have shown that people who are immunocompromised do not have the same level of protection from COVID-19 mRNA vaccines [7,8,9]. Real-world vaccine studies have shown high effectiveness in the general population, ranging from 65% to 90% against infection or mild disease and 90% to 100% against severe infection after two doses of BNT162b2 (Pfizer), AZD1222 (AstraZeneca), or mRNA-1273 (Moderna) [10,11,12,13]. However, vaccine effectiveness rates in immunocompromised populations after two doses of an mRNA vaccine are lower, at around 60% against clinical disease [9] and 77% against hospitalization [4]. Vaccine effectiveness varies widely even within the immunocompromised population, ranging from 59% (e.g., solid organ transplant) to 81% (e.g., rheumatoid arthritis) against hospitalization [4]. Moreover, there is a lack of clinical access to effective antibody testing for vaccine response in Canada and these tests have not been adequately evaluated for their ability to determine immunity or protection from COVID-19 [14]. It is therefore difficult to predict how people will respond to vaccination and their level of protection from severe COVID-19 outcomes. In addition, with the advent of new variants of concern (VOCs), such as Omicron (subvariants BA.1, BA.1.1. and BA.2), vaccine effectiveness against hospitalization has been reduced, requiring three doses in order to achieve comparable protection to two doses with the Delta (B.1.617.2) variant [15]. As of 5 April 2022, the National Advisory Committee on Immunization has also recommended a fourth dose of a COVID-19 vaccine in Canada, prioritizing adults 80 years of age and over and residents of long-term care homes and congregate living centers [16]. A discretionary recommendation is also given for adults 70–79 years of age. It is expected that the introduction of a third and fourth dose of a COVID-19 vaccine will increase the response and provide additional protection in a proportion of patients [17,18]. Those individuals not adequately protected by COVID-19 vaccination may also benefit from complementary approaches, such as pre- or post-exposure prophylaxis with monoclonal antibodies.

## 2. Risk of COVID-19 in Patients with Hematologic Malignancies

Patients with hematological malignancies, such as leukemias, myelodysplastic syndromes, myeloproliferative neoplasms, lymphomas, and multiple myeloma, have an increased risk of infections as a result of disease-related immune dysregulation [19]. In addition, many commonly used treatments may have an immunosuppressive effect, adding to the risk of infection [19]. For example, treatment with B-cell-targeting therapies, resulting in B-cell depletion and/or disruption of the B-cell receptor signaling pathway, may adversely affect the production of antibodies in response to COVID-19 vaccination [20]. B-cell recovery may also be slow in these patients; for example, in lymphoma patients, recovery was shown to remain below normal controls one year after administration of rituximab [21]. In addition, patients undergoing HSCT are at particularly high risk of immunosuppression due to impairment in innate and adaptive immunity [22]. In the case of allogeneic HSCT, patients generally need ongoing immune suppressive therapy to treat and prevent graft-versus-host disease [22]. Although data are limited, preliminary studies suggest patients with hematological malignancies who are receiving CD19-directed CAR T-cell therapy also have a reduced response to COVID-19 vaccines [23].

Seroconversion rates following COVID-19 vaccination have shown to be reduced in patients with hematologic malignancies. An Israeli study in 427 people showed that a lower proportion of those with hematologic malignancies were seropositive after COVID-19 vaccination than in an immunocompetent comparator group (75% vs. 99%; *p* < 0.001) [24]. Moreover, patients treated for hematologic malignancies (n = 164) had significantly less seropositive responses (e.g., immunochemotherapy (29%); anti-CD20 antibodies (0%); or BCL2 (25%), BTK (40%), or JAK2 (42%) inhibitors; *p* < 0.001) [24]. In addition, the prospective CAPTURE (COVID-19 antiviral response in a pan-tumor immune monitoring) study showed seroconversion rates following COVID-19 vaccination were lower in 21 patients with haematological malignancies (59%) than in those with solid tumours (85%) [25]. Overall, 81% had received chemotherapy, 48% had received targeted therapy, and 29% had received anti-CD20 therapies in the 12 weeks prior to vaccination. In addition, a study of 121 patients with non-Hodgkin’s lymphoma, including chronic lymphocytic leukemia (CLL), showed an 85-fold reduction in mean anti-SARS-CoV-2 spike immunoglobulin G–binding titers in these patients compared with healthy controls, with seroconversion occurring in only 67% of patients [26]. Overall, 47% of patients had received anti-CD20-directed therapy within one year prior to COVID-19 vaccination. Finally, a systematic review of five studies examining the response to COVID-19 vaccination in a total of 70 patients receiving CAR T-cell therapy, showed a low cumulative humoral response rate of 31% [23].

A number of studies have shown patients with hematologic malignancies are at increased risk of severe outcomes following infection with COVID-19. A study by Mittelman et al. showed that among 32,516 vaccinated patients with hematologic malignancies, the relative risk for COVID-19 infection (1.60; 95% CI, 1.12–2.37) and hospitalization (3.13; 95% CI, 1.68–7.08) was significantly greater, compared with matched controls [27]. Overall, 5107 (15.9%) patients were receiving active treatment for a hematological malignancy. In addition, a study examined data from the VISION Network, which included 20,101 immunocompromised patients aged ≥ 18 years with COVID-19-like illness discharged from 187 hospitals across nine states in the United States [4]. Results showed that in patients with hematological malignancies, vaccine effectiveness against hospitalization was around 74% (95% CI: 62%, 83%), versus 90% (95% CI: 89%, 91%) in immunocompetent patients who had received two doses of an mRNA vaccine two weeks prior to hospitalization. In addition, a study in 2767 patients with non-Hodgkin lymphoma showed those receiving active treatment given within 30 days of COVID-19 diagnosis (n = 195) had more severe outcomes than those not receiving treatment (OR 1.4; 95% CI 1.0, 2.0) [28]. Two large multicenter retrospective studies have also reported high rates of severe COVID-19 disease and mortality in both untreated as well as treated patients with CLL [29,30]. In one of the studies [26], survival rates were not associated with active treatment, whereas in the second study [27], significantly more patients (91/151, 60.3%) in the severe COVID-19 group were off treatment within the last year or had never received treatment for CLL compared with less severe cases (15/39, 38.5%) (*p* < 0.05). Finally, The Center for International Blood and Marrow Transplant Research (CIBMTR) found that severe COVID-19 disease requiring mechanical ventilation occurred in 45/318 (14%) of HSCT recipients and thirty-day survival was around 67% [22]. Given the increased risk of severe COVID-19 outcomes in patients with hematologic malignancies, broader protection is needed beyond vaccination.

## 3. Prophylaxis against COVID-19 Infection

Several monoclonal antibodies have been approved by Health Canada for the treatment of COVID-19. These include sotrovimab, casirivimab–imdevimab (REGEN-COV), and bamlanivimab, which are approved for the treatment of mild to moderate COVID-19 disease in adults who are at high-risk for progressing to hospitalization and/or death [31,32,33] (Table 1). More recently, monoclonal antibodies have also been examined as pre-exposure and post-exposure prophylaxis. When used as prophylaxis, the therapy is given before or after exposure to COVID-19, but prior to a positive COVID-19 test result. To date, only the tixagevimab–cilgavimab (Evusheld) regimen is approved by Health Canada as prophylaxis for COVID-19 [34] (Table 1). However, in addition to tixagevimab–cilgavimab [6], the U.S. Food and Drug Administration (FDA) has also granted Emergency Use Authorization (EUA) for the monoclonal antibody combinations casirivimab–imdevimab [35] and bamlanivimab–etesevimab [36] as prophylaxis in select individuals at high risk of progression to severe COVID-19. Results of key trials leading to FDA approval for the three prophylactic agents against COVID-19 are presented in Table 2.

### 3.1. Casirivimab–Imdevimab

Casirivimab and imdevimab are two neutralizing monoclonal antibodies that bind non-competing epitopes on the receptor–binding domain of the SARS-CoV-2 spike protein, blocking virus entry [41]. The casirivimab–imdevimab combination was first approved by Health Canada as treatment for COVID-19 infection. Health Canada approval of casirivimab–imdevimab as treatment for COVID-19 infection in individuals at high risk of hospitalization or death, was based on the REGN-COV2 study published in 2020, which demonstrated a 70.4% reduction in the number of patients with a COVID-19-related hospitalization or all-cause death versus placebo following a single intravenous dose (1200 mg) of casirivimab–imdevimab (*p* < 0.0024) [33,42]. Subsequently, casirivimab–imdevimab was examined in a Phase 3 trial versus placebo as post-exposure prophylaxis against COVID-19 [37]. The study evaluated a single subcutaneous dose (1200 mg) of casirivimab–imdevimab for prevention of COVID-19 in 1505 household contacts of individuals infected with SARS-CoV-2. However, only 0.5% of the study population had immunosuppressive disease. An 81.4% risk reduction in confirmed symptomatic COVID-19 cases was observed with casirivimab–imdevimab compared to placebo at day 29 in cases who were RT-PCR negative and seronegative at baseline (the primary analysis population). Overall, rates of adverse events were similar between groups (20.2% vs. 29.0% for casirivimab–imdevimab and placebo, respectively). Adverse events that occurred with a frequency of at least 2% with casirivimab–imdevimab included symptomatic and asymptomatic COVID-19, headache, and injection-site reaction.

As a result of the post-exposure prophylaxis data, casirivimab–imdevimab was granted FDA EUA approval for post-exposure prophylaxis of COVID-19 in individuals who are at high risk for progression to severe COVID-19, including hospitalization or death [35]. However, casirivimab–imdevimab has not yet been approved for this indication in Canada.

Data from neutralization assays have shown casirivimab–imdevimab is unlikely to be active against the B.1.1.529/BA.1 (Omicron; South Africa origin) variant [35]. The potential of casirivimab and of imdevimab to mediate viral entry was assessed in immune cell lines co-incubated with recombinant vesicular stomatitis virus (VSV) virus-like particles (VLP) pseudotyped with SARS-CoV-2 spike protein at concentrations of monoclonal antibodies down to approximately 10-fold below the respective neutralization EC50 values. Casirivimab and imdevimab, individually (>1732-fold reduction and >754-fold, respectively) and together (>1013-fold), demonstrated reduced neutralization activity against VLP pseudotyped with the full spike protein sequence of the B.1.1.529/BA.1 (Omicron; South Africa origin) lineage. As a result, this combination is not recommended by the FDA in regions where B.1.1.529/BA.1 is predominant.

### 3.2. Bamlanivimab–Etesevimab

Bamlanivimab and etesevimab are two potent neutralizing monoclonal antibodies that target the surface spike glycoprotein of SARS-CoV2 that mediates viral entry into host cells [43]. Health Canada approval of bamlanivimab monotherapy as treatment for COVID-19 in individuals at high risk of hospitalization or death was based on initial data for the monotherapy arms of the phase II/III Blocking Viral Attachment and Cell Entry with SARS-CoV-2 Neutralizing Antibodies (BLAZE-1) trial [44]. BLAZE-1 examined bamlanivimab as monotherapy or in combination with etesevimab, versus placebo in reducing COVID-19 viral load in 577 infected participants [33,44]. Compared with placebo, only the combination therapy significantly reduced viral load [−0.57 (*p* = 0.01)]. However, a lower proportion of bamlanivimab-treated subjects progressed to COVID-19-related hospitalization or emergency room visits for each of the monotherapy doses and for the combination arm, compared to placebo-treated subjects. The proportion of patients with COVID-19–related hospitalizations or emergency department visits was 5.8% (9 events) for placebo, 1.0% (1 event) for 700 mg, 1.9% (2 events) for 2800 mg, 2.0% (2 events) for 7000 mg, and 0.9% (1 event) for combination treatment. Although the FDA initially granted EUA authorization for bamlanivimab monotherapy, it was later revoked based on the increase in COVID-19 variants that are resistant to bamlanivimab monotherapy. Authorization was granted instead for the combination with etesevimab [43]. However, to date, Health Canada approval remains only for treatment of COVID-19 with bamlanivimab monotherapy.

Subsequently, bamlanivimab monotherapy was examined in the phase III BLAZE-2 trial as monotherapy for post-exposure prevention of COVID-19 in residents and staff of nursing facilities following a confirmed case of COVID-19 infection [38]. Those with a positive baseline SARS-CoV-2 RT-PCR test were included in the treatment population (N = 132) and those with a negative test were included in the prevention population (N = 966). A total of 575/966 participants in the prevention population were considered to be at high risk of severe COVID-19 outcomes due to factors including but not limited to age ≥ 65 years with body mass index ≥ 35, chronic kidney disease, type 1 or type 2 diabetes, immunosuppressive disease or receiving immunosuppressive treatment, and other comorbidities. Bamlanivimab significantly reduced the incidence of COVID-19 in the overall post-exposure population compared with placebo (8.5% vs. 15.2%; OR 0.43; *p* < 0.001). The rate of participants with adverse events was similar in both groups (20.1% vs. 18.9% in the bamlanivimab and placebo group, respectively). The most common adverse events in the bamlanivimab group were urinary tract infection (2%) and hypertension (1.2%).

As a result of the data from BLAZE-2, the FDA granted EUA for bamlanivimab and etesevimab administered together for post-exposure prophylaxis of COVID-19 in individuals who are at high risk of progression to severe COVID-19, including hospitalization or death [36]. The authorization was for the combination regimen, despite the fact that the BLAZE-2 study examined bamlanivimab as monotherapy. The FDA justified authorization for combination therapy based on the BLAZE-1 combination data that demonstrated a statistically significant reduction in progression of severe COVID-19, including hospitalization or death. However, bamlanivimab–etesevimab has not yet been approved for this indication in Canada.

Data from neutralization assays have shown bamlanivimab and etesevimab are also unlikely to be active against the B.1.1.529/BA.1 (Omicron; South Africa origin) variant [36] Pseudotyped virus-like particles expressing the spike protein from the B.1.1.529/BA.1 show reduced susceptibility to bamlanivimab alone (>1465-fold), etesevimab alone (>616-fold), and bamlanivimab and etesevimab together (>2938-fold) [36]. Therefore, bamlanivimab–etesevimab is also not recommended by the FDA in areas where the Omicron strain is predominant.

### 3.3. Tixagevimab–Cilgavimab

Tixagevimab–cilgavimab comprises two fully human extended half-life SARS-CoV-2–neutralizing antibodies that bind distinct epitopes of the viral spike protein receptor binding domain [39,40]. Unlike casirivimab–imdevimab and bamlanivimab, tixagevimab-cilgavimab is not approved by Health Canada or the FDA for the treatment of COVID-19. Instead, tixagevimab–cilgavimab is approved as pre-exposure prophylaxis against COVID-19 in individuals who are not currently infected and who have no recent exposure to an infected individual [6,34]. The primary data supporting the pre-exposure prophylaxis recommendation are from the phase III PROVENT trial [39,40]. PROVENT included 5197 unvaccinated people with either an increased risk of inadequate response to vaccination or increased risk of COVID-19, exposure randomized to two intramuscular injections (150 mg each) of tixagevimab and cilgavimab given sequentially for symptomatic COVID-19 prevention. Of note, about 78% had baseline comorbidities or characteristics associated with an increased risk for severe COVID-19 (e.g., obesity, diabetes, cardiovascular disease), but only 7% had active cancer or a history of cancer and only 3% had received immunosuppressive medications [2]. The primary endpoints were first case of symptomatic COVID-19 positivity and safety 183 days post administration [39,40]. After a median of 83 days, tixagevimab–cilgavimab reduced the risk of developing symptomatic COVID-19 by 76.7% (95% CI 46.0, 90.0) versus placebo (*p* < 0.001). Tixagevimab–cilgavimab was well tolerated with similar rates of adverse events in each group (35.3% vs. 34.2% in the tixagevimab–cilgavimab and placebo groups, respectively). The most common adverse event of special interest was injection-site reaction, which occurred in 2.4% of participants in the tixagevimab–cilgavimab group versus 2.1% in the placebo group. There was also a slight numerical increase in the frequency of cardiac disorders with tixagevimab–cilgavimab versus placebo (0.7% vs. 0.3%, respectively). After a median follow-up of 6 months, the relative risk reduction was 82.8% (95% CI: 65.8, 91.4) for symptomatic illness, with 11/3441 (0.3%) events in the tixagevimab–cilgavimab arm and 31/1731 (1.8%) events in the placebo arm [6,34,40].

In the era of Omicron spread, early data suggest tixagevimab–cilgavimab maintains at least partial efficacy against this variant and could provide additional protection for high-risk patients [6]. The neutralization activity of tixagevimab–cilgavimab against the Omicron subvariants (BA.1, and BA.1.1 [BA.1+R346K]) versus the reference strain decreases 12- to 424-fold and consequently the duration of protection is not known and is likely reduced. Conversely, the neutralization activity against the Omicron BA.2 subvariant versus the reference strain is minimally impacted. Due to decreased neutralization activity of tixagevimab–cilgavimab against the Omicron subvariants BA.1 and BA.1.1 (BA.1+R346K), the current Health Canada recommendation is to consider increasing the dose from 300 mg to 600 mg in regions where the BA.1 and BA.1.1 variants are circulating.

## 4. Canadian Perspective

In view of the ongoing COVID-19 risk, there is an important need for Canadians to have access to effective pre- and post-COVID exposure monoclonal antibody prophylaxis. Given the widespread prevalence of the Omicron variant in Canada, the use of casirivimab–imdevimab or bamlanivimab–etesevimab antibody combinations is unlikely to be successful in reducing symptomatic infections [6,35,36]. However, there is evidence of at least partial protection against the Omicron variant with the tixagevimab–cilgavimab combination, justifying its use in high-risk populations in Canada. With the lifting of restrictions across the country, it is crucial to protect patients at highest risk of serious COVID-19 outcomes, whereas rates of Omicron remain high. Moreover, fatigue from social isolation related to physical distancing makes it harder for vulnerable patients to continue to isolate and protect themselves by avoiding others. Use of tixagevimab–cilgavimab in these vulnerable populations may therefore be a valuable tool to provide better protection against serious outcomes from COVID-19. However, the possible signal for an increase in cardiac disorders with tixagevimab–cilgavimab and the risk/benefit of therapy needs to be carefully discussed with any patient who has active cardiac disease.

Identification of suitable patients who may benefit from such prophylaxis is paramount. Unfortunately, studies examining the effectiveness of monoclonal antibodies as prophylaxis against COVID-19 have not included a significant number of patients with hematological malignancies or examined the efficacy in this subgroup. However, it is clear that these patients are in need of additional protection from COVID-19 infection given the immunosuppression associated with their disease and related treatment. According to the American Society for Hematology (ASH), high-risk criteria relevant to hematology patient populations include immunosuppressive disease or immunosuppressive treatment (post-allogeneic transplantation, active or recent chemotherapy or immunotherapy, active malignancy) or sickle cell anemia, and patients unlikely to respond optimally to vaccination or not fully vaccinated [45]. According to the National Comprehensive Cancer Network (NCCN) guidelines on COVID-19 Vaccination and Pre-exposure Prophylaxis, patients with hematologic malignancies (including those receiving HSCT or engineered cellular therapy) are more likely to have inadequate responses to COVID-19 vaccination and are at highest risk of major COVID-19 complications. The committee agreed that a reasonable option is to prioritize these patients with hematological malignancies for tixagevimab–cilgavimab [46]. Health Canada has approved the use of tixagevimab–cilgavimab as pre-exposure prophylaxis in individuals who are immunocompromised and unlikely to mount an adequate response to COVID-19 vaccination or where vaccination is not recommended [34].

In Canada, quantitative antibody testing for protective COVID-19 antibodies is not easily available and is not generally recommended. These tests have not been adequately evaluated for their ability to determine immunity or protection from COVID-19, and therefore should not be solely relied on to identify patients at highest risk of acquiring COVID-19 infection.

It is likely that for vulnerable individuals, pre-emptive therapy with tixagevimab–cilgavimab will prevent significant COVID-19-related illness. There is presently a lack of cost-effectiveness analyses for tixagevimab–cilgavimab, as for other COVID-19 therapies made available through the federal government to the provinces. However, this approach may be cost-effective, by preventing severe outcomes and reducing hospitalization, thereby allowing patients a more rapid return to work. Importantly, this added protection may improve quality of life by allowing patients to more confidently return to activities of daily living that include increased contact with others, such as social interaction.

## Figures and Tables

**Table 1:** Health-Canada-approved monoclonal antibodies for the treatment and prevention of COVID-19.

Monoclonal Antibody	Indication	Age Range	Timing of Administration
**Sotrovimab [31]**	Treatment of mild to moderate COVID-19 who are at high risk for progressing to hospitalization and/or death	Adults Adolescents ≥ 12 years of age and weight ≥ 40 kg	As soon as possible after symptom onset *
**Casirivimab-imdevimab [32]**	Treatment of mild to moderate COVID-19 who are at high risk for progressing to hospitalization and/or death	Adults Adolescents ≥ 12 years of age and weight ≥ 40 kg	As soon as possible after exposure
**Bamlanivimab [33]**	Treatment of mild to moderate COVID-19 who are at high risk for progressing to hospitalization and/or death	Adults Adolescents ≥ 12 years of age and weight ≥ 40 kg	As soon as possible after symptom onset
***Tixagevimab-cilgavimab*** **[34]**	Pre-exposure prophylaxis in individuals who are immunocompromised or where vaccination is not recommended	Adults Adolescents ≥ 12 years of age and weight ≥ 40 kg	Prior to exposure

* Study treated only patients within 5 days of symptom onset.

**Table 2:** Key Phase 3 Trials of Pre- and Post- COVID-19 Prophylaxis.

Study	Population	Treatment	Efficacy Results	Safety Results
**O’Brien et al., 2021 [37]**	Close household contacts of a SARS-CoV-2-infected index (N = 1505)	Casirivimab–Imdevimab (600 mg of each SC) vs. Placebo	**Primary Endpoint: Proportion of seronegative participants who developed symptomatic COVID-19** Relative Risk Reduction: 81.4%; *p* < 0.001 Progression to symptomatic disease: Casirivimab–Imdevimab: 11/753 (1.5%) vs. Placebo: 59/752 (7.8%)	AEs: casirivimab–imdevimab: 20.2% vs. placebo: 29.0% Most common AEs: symptomatic and asymptomatic COVID-19, headache, injection-site reaction No dose-limiting toxic effects were reported
**BLAZE-2 [38]**	Residents and staff at U.S. skilled nursing and assisted living facilities with at least 1 confirmed SARS-CoV-2 index case and negative at baseline for SARS-CoV-2 infection and serology (N = 966 from prevention cohort)	Bamlanivimab (4200 mg IV) vs. Placebo	**Primary Endpoint: Incidence of COVID-19** COVID-19 incidence: 8.5% vs. 15.2%; OR 0.43; *p* < 0.001 Absolute Risk Difference: −6.6 (95% CI, −10.7 to −2.6)	AEs: bamlanivimab: 20.1% vs. placebo: 18.9% Most common AEs: Urinary tract infection: bamlanivimab: 2% vs. placebo: 2.4% Hypertension: bamlanivimab: 1.2% vs. placebo: 1.7%
**PROVENT [6,39,40]**	Unvaccinated adults without prior SARS-CoV-2 infection Increased risk of either inadequate response to vaccination or SARS-CoV-2 exposure (N = 5197)	Tixagevimab–Cilgavimab (150 mg of each as 2 IM) vs. Placebo	**Primary Endpoint: Incidence of first case SARS-CoV-2 RT-PCR–positive symptomatic illness** *3-Month Follow-Up* Relative Risk Reduction: 76.7% (95% CI 46.0, 90.0) vs. placebo (*p* < 0.001) *6-Month Follow-Up* Relative Risk Reduction: 82.8% (95% CI: 65.8, 91.4)	AEs: tixagevimab–cilgavimab: 35.3% vs. Placebo: 34.2% Most common AE: injection-site reaction: tixagevimab–cilgavimab: 2.4% vs. placebo: 2.1% Cardiac disorders: tixagevimab–cilgavimab: 0.7% vs. placebo: 0.3%

AE, adverse event; CI, confidence interval; IM, intramuscular; IV, intravenous; OR, odds ratio; SC, subcutaneous.
